# A CRISPR View of Biological Mechanisms

**DOI:** 10.15190/d.2016.16

**Published:** 2016-12-31

**Authors:** Eduardo Martinez, Lilia Sanchez, Neftali Vazquez, Rebecca Marks, Raechel Cedillo, Christa Respondek, Martin Holguin, Michael W. Persans, Megan Keniry

**Affiliations:** Department of Biology, University of Texas Rio Grande Valley, 1201 W. University Dr., Edinburg, TX 78539, USA

**Keywords:** CRISPR, Cas, Review

## Abstract

A decade ago, only six manuscripts would be found on a PubMed search for “CRISPR,” compared to 2,011 manuscripts in 2016. The purpose of this review is to discuss this emergent technology that has revolutionized molecular biological research in just a few years. Endogenous CRISPR mechanisms are harbored by bacteria and archaea as an adaptive defense system that targets foreign DNA from viruses and plasmids. CRISPR has been adapted as a genome editing tool in a plethora of organisms ranging from yeast to humans. This tool has been employed to create loss of function mutations, gain of function mutations, and tagged alleles in a wide range of settings. CRISPR is now extensively employed for genetic screens. CRISPR has also been adapted to study transcriptional regulation. This versatile and relatively facile technique has, and will be, tremendously impactful in research areas such as biomedical sciences, agriculture, and the basic sciences.

## SUMMARY

1. *Introduction*
2. *Endogenous Roles for CRISPR in Bacteria and Archaea*
3. *Adaptation of CRISPR Cas9 as a Genome Editing Tool*
4. *Novel Applications of CRISPR*
  - *4.1 CRISPR Employed to Study Transcriptional Regulation*
  - *4.2 CRISPR Employed to Study Epigenetics*
  - *4.3 CRISPR Employed in Genome Imaging*
  - *4.4 CRISPR Employed in Tracking mRNA*
  - *4.5 CRISPR Employed in Lineage Tracing*
  - *4.6 CRISPR Screens*
5. *Ethical Issues of CRISPR Technology*
6. *Future Directions for CRISPR Technology*
7. *Conclusions*

## 1. Introduction

Both domains of prokaryotes harbor adaptive immunological responses to fend off foreign DNAs that enter the cell from viruses and plasmids^1,2^. This foreign DNA is recognized as such at least in part by harboring a short nucleotide sequence termed a PAM (Protospacer adjacent motifs)^3-5^. Pieces of the invading DNA are incorporated into a specialized prokaryotic CRISPR (Clustered Regularly Interspaced Short Palindromic Repeats) locus^4^. Transcription of the CRISPR locus leads to a molecular machine that targets the foreign viral and plasmid DNAs with an exonuclease such as Cas9^6^.

Similar to restriction enzymes and certain reporter genes, CRISPR technology has been adapted from prokaryotes to be a very powerful molecular biological tool in diverse biological systems including yeast, flies, worms, mice, plants and human cells^2,7-29^. Heterologous CRISPR systems were initially utilized as a genome editing tool to modify genomic architecture and accordingly function^11,12,15,16,28,29^. However, CRISPR can also be utilized in functional assays to examine transcriptional regulation and other biological processes^30-32^.

The utilization of CRISPR as a versatile tool has revolutionized molecular biology and driven it into a new era. Genetic analyses that were nearly technically impossible in the past are becoming commonplace^2,3,7-32^.This review discusses CRISPR as one of the most important scientific discoveries of the 21^st^ century.

## 2. Endogenous Roles for CRISPR in Bacteria and Archaea

**Figure 1:**
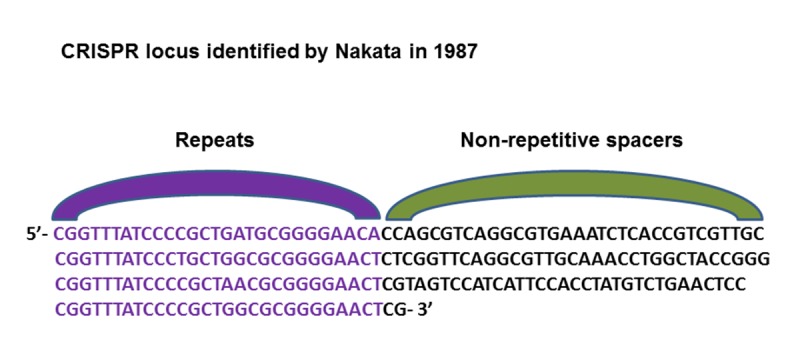
Unique Genomic Locus noticed in 1987 would later be realized as a CRISPR locus Nakata et al.^33^ discovered repetitive sequences separated by non-repetitive spacer sequences on the 3’ end of the IAP gene in E. Coli in 1987. This striking arrangement of DNA sequences later became known as CRISPR (clustered regularly interspaced short palindromic repeats).

In 1987 clusters of repeated DNA with dyad symmetry were noticed in Japan by Atsuo Nakata^33^. Nakata was examining the *IAP* gene in Gram-negative *Escherichia coli (E. coli)* when unique repetitive sequences were uncovered on the 3’ end of the gene. **Figure 1**shows a schematic of the repeated DNA with dyad symmetry for the *IAP *gene^33^. Nakata identified a consensus sequence: 5’-CGGTTTATCCCCGCT –GG -or- AA- CGCGGGGAACTC-3’ that was repeated five times. In between each repeat was a distinct spacer that was non-repetitive and about 31 nucleotides long. Finding repetitive sequences within a genome was not at all surprising as almost all prokaryotic or eukaryotic genomes previously examined have had repeat sequences^1,2,34^. However, the newly found repeats stood out as being separated by non-repetitive sequences or spacers^33^. This arrangement of DNA sequences later became known as CRISPR, clustered regularly interspaced short palindromic repeats^1^.

Not limited to *E. coli*, CRISPR was described in gram positive bacteria in 1991 and archaea in 1993^1,34^. By 2002, CRISPR elements were found in all archaeal genomes examined and about 40% of bacterial genomes^1^. Furthermore, associated genes were universally located in the proximity of CRISPR loci; these associated genes were termed CRISPR associated sequences or Cas genes in 2002^1^. The location of the Cas genes hinted at their potential regulatory mechanisms^1^. By 2005 it was recognized that the spacer (non-repetitive sequences) were very highly homologous to viral and plasmid DNAs (invading DNAs for bacteria)^35^. Then, in 2007 Horvath’s seminal work demonstrated that bacteria incorporated the foreign DNA into the CRISPR locus upon phage infection, leading to phage resistance^4^. Mutation of these newly incorporated sequences abrogated resistance^4^. This work also demonstrated that mutation of Cas5 led to a loss of resistance even if the CRISPR locus was already modified to harbor the foreign DNA, whereas Cas7 was dispensable for resistance once the CRISPR locus was modified^4^. This work linked the spacer DNA to adaptive bacterial immunity and began to delineate the specific roles of Cas proteins in this process.

The precise mechanisms utilized by CRISPR are still an active area of investigation. The spacer (non-repetitive sequences) are transcribed into small RNAs (crRNAs) and in combination with another RNA (trans activating crRNA or tracrRNA) form a complex with Cas proteins to target sequences that are complimentary to the guide RNA^6,36-42^. There are numerous types of Cas mechanisms^2,28,37-41^. Type I systems employ Cas5 or Cas6 to process the pre-CRISPR RNA into a crRNA, which will be utilized by Cas3 to target DNAs^43^. The Type II systems employ Cas9 to target genes; the processing events leading to mature crRNA are not completely elucidated^43^. Cas9-containing Type II systems are commonly used in heterologous systems for genome editing^43^. Type III systems employ Cas6 to process pre-CRISPR RNA to crRNA, which functions in concert with Csm/Cmr to target foreign DNAs^43^. Short DNA sequences adjacent to the non-repetitive sequences (PAMs) have a dual role of distinguishing self from non-self and in recognition by the components of the CRISPR machinery such as the Cas9 exonuclease^44-48^. A typical PAM sequence is 2-4 base pairs long (such as -NGG) and is not found in the host bacterial genome thereby establishing selectivity for invading DNAs^48^.

Each CRISPR system employs a specific nuclease. For example, Type II systems utilize Cas9 endonucleases that contain RuvC-like and HNH domains that cleave DNA to produce double strand breaks^3,14,25,30,46,47^. Transcription of the *CRISPR* locus produces a pre-crRNA, which will be processed and pair with a tracrRNA, thereby enabling processing and incorporation into a Cas9-containing complex. Hybridized crRNA::tracrRNA complexes serve as a guide for Cas9 to cleave foreign DNA in a sequence-specific manner^6,29,41^.

The diversity of CRISPR systems is not at all limited to the TYPE I, II or III Systems^*^43,49-51^*^. In an analysis of 100 *E. coli* strains, tremendous diversity of CRISPR loci (and even systems) was discovered^50^. Repeat sequences are typically 21 to 48 base pairs in length whereas the spacers are typically between 26 and 72 base pairs in length^49,50^. Archaeal CRISPR loci tend to be larger than bacterial loci^52^. CRISPR Cas systems in archaea have been found to have an almost 100% immunity in instances when the spacer sequence was identical to the target sequence^52^. Adaptive immunity is passed on by a “Lamarckian inheritance” principle, some bacteria and archaea have immunity to something they have never encountered during their lifespan^53,54^.

The CRISPR defense process can be broken up into three stages. The first stage is adaption, which is when new spacers are added to the CRISPR locus post foreign DNA exposure^4,6,51,54^. The second stage is expression of the locus. The *CRISPR* locus is transcribed into a long precursor RNA^4,6,51,54^. This precursor is then processed into crRNA by Cas proteins and accessory components^43^. The final stage, target nucleic acids are recognized and destroyed by crRNA combined with traRNA and Cas proteins^55^. When CRISPR was first discovered in bacteria it was thought to be a genetic abnormality, now almost 20 years later it is known to be a prokaryotic cell’s adaptive immunity to foreign DNA threats^2,33^. Interestingly, CRISPR is also endogenously used in prokaryotic gene regulation^56^.

## 3. Adaptation of CRISPR as a Genome Editing Tool

CRISPR technology has the ability to efficiently modify endogenous genes in various species and cell types, and may even serve as potential therapy for genetic diseases. Before CRISPR, genomic alterations were limited to certain model organisms such as yeast and mice^57,58^. Through heterologous CRISPR technology, genes may be mutated via non-homologous end joining (NHEJ) or homologous recombination mechanisms in a plethora of organisms^2,55^. CRISPR can be used to tag genes with GFP for visualization or other tags for complex purification^59^.

Heterologous CRISPR systems typically employ Cas9 complexes and synthetic guide RNAs (sgRNAs) that are hybrids of the tracrRNA and crRNA found in endogenous CRISPR complexes^25^. The sgRNAs localize Cas9 to genes of interest leading to the formation of a double strand break^25^. Next, the double strand break is resolved by NHEJ or homology-directed repair (HDR)^25^. NHEJ is used to introduce insertion or deletion mutations, which may vary in length and may shift the reading frame of a coding sequence^25^. Homology-directed repair is used more for the specific point mutation or insertion via recombination of desired sequence with a donor template^25^. Heterologous systems vary tremendously in efficiency depending on the context^60,61^. For example, efficiencies of 1-4% were observed in HAP1 cells and 2-22% in U2OS osteosarcoma cells^15,16,43,62^. One other pitfall of employing CRISPR for mutagenesis is the issue of off-target effects^62^. To minimize off-target effects, researchers have engineered the Cas9 D10A mutant, which harbors a mutant RuvC domain, leading to a nickase^10^. The Cas9 HNH domain can also be inactivated (H840A mutant) to generate a nickase^15^. Cas9 D10A, H840A double mutants lack the ability to cleave DNA, but retain DNA binding^15^. Mutagenesis reactions that employ the Cas9 D10A (or Cas9 H840A) nickase utilize 2 sgRNAs that are in close proximity to separately target the top and bottom strand of a gene of interest, thereby increasing mutational specificity^9^.

## 4. Novel Applications of CRISPR

CRISPR Cas9 technology has remarkable flexibility as a tool for not only genome editing, but in other areas such as investigating transcriptional control, epigenetic regulation and genomic imaging^25,30,59^. There are countless applications for CRISPR technology, which are continuing to develop at an impressive rate.

### 4.1 CRISPR Employed to Study Transcriptional Regulation

Beyond its use as a gene editing tool, CRISPR Cas9 technology was repurposed as an alternative to RNAi^63^. The Cas9 protein was converted into a nuclease dead protein (dCas9) by disrupting its endonuclease domains RuvC and HNH^63^. Using guide RNA to direct its binding to specific genes, dCas9 was able to effectively repress gene expression in bacteria by physically blocking RNA polymerase access to genes of interest^63^. This provides an alternative to RNAi, which induces knockdowns via destruction of mRNA, whereas CRISPR provides regulation at a transcriptional level^24^. This system has been shown to be highly tunable as well. The position of the sgRNA can be easily modified and determines the strength of its repressive effects, sometimes in the range of 1000-fold repression^64^. A distinct advantage the use of CRISPR has over RNAi is CRISPR dCas9 can activate transcription as well. By fusing the dCas9 complex with a C-terminal VP64 acidic transactivator, there was a large increase of VP64 in HEK293 cells^65^. These results also provide an alternative to laborious engineering of transcription factors that are commonly used for controlling gene expression^65^. CRISPR also seems to be less prone to off-target effects than RNAi which has been becoming a growing concern as a tool for gene study. RNAi suffers from a limited sequence complementation, which can lead to off-target silencing and phenotypes^66^. Additionally, RNAi is documented to induce interferon responses leading to artefactual phenotypes^67-69^.

### 4.2 CRISPR Employed to Study Epigenetics

Prior to 2013, the most common tools in epigenomic studies are zinc finger nucleases (ZFNs) and TALENs (Transcription-Activator-Like effector nucleases)^66^. Recently however, CRISPR has steadily become more versatile and has expanded into epigenomic studies, providing another alternative mechanism with its own benefits^66^. Zinc Finger Nucleases are limited by high costs and the demands for more effort in the creation of proteins^66^. TALENs also suffer from the difficulty of custom protein creation; however, it is more streamlined and in some cases, can outperform CRISPR as it suffers fewer issues from off-target effects making it still a viable option today^19,23,66^. CRISPR benefits from the ease of designing sgRNAs, high efficiency and specificity^66^. CRISPR technology can lead to off-target effects; however, protocols are rapidly being improved, such as implementing shorter sgRNAs that lack the areas that allow mismatch or using mutant forms of Cas9^70^. Due to CRISPR having easily producible sgRNA constructs and specificity gives it better scalability to alter multiple sites in the genome, allowing simultaneous gene editing in a single organism^31,32,63,71^.

Recent studies have shown that CRISPR can be used as a highly specific epigenomic editing tool. The modular protein p300 contains a catalytic histone acetyltransferase (HAT) core domain that acetylates histones^72^. By fusing dCas9 to this core domain, it was shown that CRISPR could significantly and precisely induce transcription when targeting endogenous promoters of *IL1RN*, *MYOD* and *OCT4* in human HEK293T cells^30^.

Another study showed that fusing dCas9 with Krüppel associated box (KRAB) domain, a domain that recruits a heterochromatin forming complex that cause histone methylation and de-acetylation, and targeting HS2 enhancer in the globin locus control region (LCR) would effectively disrupt the expression of globin genes in erythroid cells with nearly perfect specificity^73^. These studies show that CRISPR is a viable tool for epigenomic study that has already provided suitable alternatives to other current methods employed.

### 4.3 CRISPR Employed in Genome Imaging

CRISPR Cas9 systems have also been adapted to tag cells with fluorescent proteins for live cell imaging. Fluorescent live cell imaging is a powerful tool for investigating the contribution of cellular processes to functional genome output. Traditional methods such as fluorescent in situ hybridization (FISH), require sample fixation and cannot be used for live cell imaging^14^. By fusing Cas9 proteins with fluorescent tags, CRISPR provides a target specific, fast and convenient alternative to traditional imaging without the use of disruptive treatments^63^.

Genome imaging studies have already led to groundbreaking insights into telomerase function^74^. Previous work using FISH or SNAP-tagged TERT in fixed cells led to the conclusion that telomerase only associated with telomeres during S-phase and with Cajal bodies during the rest of the cell cycle. However, utilizing the Cas9 system endogenous telomerase was tagged with a red fluorescent protein, Cajal bodies with a blue fluorescent protein and telomeres with a photoswitchable green/ red fluorescent protein to visualize telomerase localization^74^. Researchers concluded that that telomerase freely diffused through the nucleus while a small subset associated with telomeres and Cajal bodies at any given time. Telomerase was also observed having two types of interactions with telomeres: short-lived probing interactions and occasional static interactions that could last up to 8 minutes^74^. This form of imaging overcame the previous hurdle present in live cell imaging of telomerase recruitment due to the low abundance of this enzyme. Overexpression of telomerase was not a solution, because exogenous expression leads to occupancy on all telomeres, not reflective of endogenous dynamics^75,76^.

### 4.4 CRISPR Employed in Tracking mRNA

Methods that address the need for tracking mRNA have also been developed. Existing methods that target RNA are Pumilio and FBF homology (PUF) proteins and the use of RNA aptamers. PUF proteins fluoresce upon binding to target mRNAs but must be redesigned and be microinjected to reduce excessive background signal^77^. This process is labor intensive and requires experience to prevent cell disruption^78^. The use and development of mRNA aptamers have also been limited by time and expense constraints^79,80^. CRISPR provides a simpler method of RNA targeting without the need for extensive protein libraries or genetic manipulations. The recent development of RCas9 has allowed for the tracking of mRNA in live cells with sgRNAs that target mRNA and the addition of PAMmer oligonucleotide containing a PAM site. This takes advantage of the targeting mechanism of Cas9 by utilizing mismatched PAM sequence to target certain RNAs exclusively. Researchers have demonstrated that this RCas9 system could recognize *GAPDH*, *ACTB*, *CCNA2* and *TFRC* mRNAs in live cells^77^.

### 4.5 CRISPR Employed in Lineage Tracing

Recently CRISPR has demonstrated its use in lineage tracing. This involves following marked cells and their descendants through development and utilizes various methods such as marking cells with dyes and enzymes, cross-species transplantation of cells and insertion of foreign DNA^81,82^. These methods however are limited by large-scale reconstruction of cell lineages or are expensive as they require whole genome sequencing. Researchers developed genome editing of synthetic target arrays for lineage tracing (GESTALT) as a method that utilizes CRISPR Cas9 to accumulate combinatorically diverse mutations that build up within edited barcodes over generations in HEK293T cells and zebrafish. By using the patterns of mutations, lineage trees could be inferred using maximum parsimony^83^.

### 4.6 CRISPR Screens

**Figure 2:**
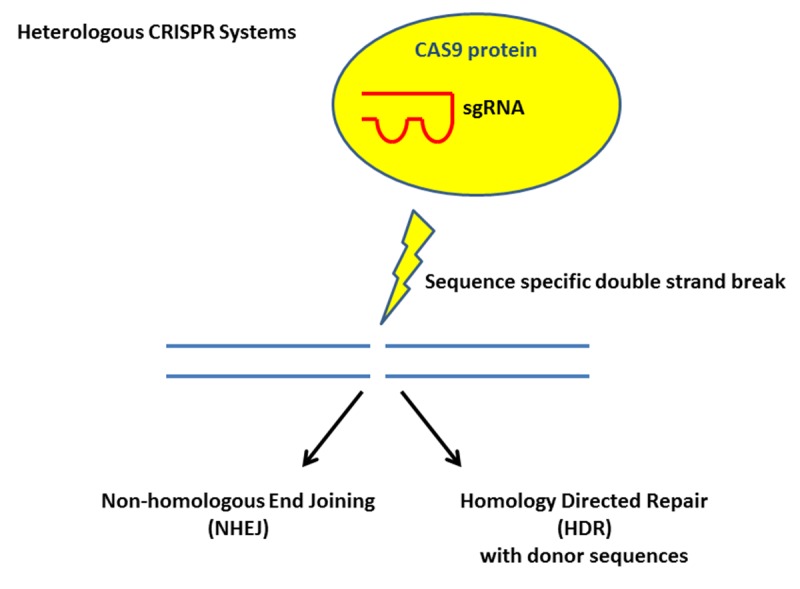
Scheme for Heterologous CRISPR Schematic demonstrates how the Cas9 exonuclease forms a complex with the synthetic guide RNA (sgRNA) in order to create double strand breaks in a sequence-specific manner. These double strand breaks are resolved by Non Homologous End Joining, which is error-prone commonly leading to insertion or deletions or Homology Directed Repair (HDR) with donor sequences to insert sequences of interest at the break site.

Heterologous CRISPR systems (**Figure 2**) as a genome editing tool has been employed to develop large scale screening techniques aimed at investigating multiple gene functions in both cell culture and *in vivo* environments^18,63^. The ease of creating specific libraries of sgRNAs at an economical rate and infecting cells from different mammalian cells lines^84^, melanoma and stem cell lines^85,86^, and *Drosophila* cells^87^ have led to various findings that are important to genomic studies^*88*^. Innovations in the CRISPR technology are constantly improving screening techniques. One such advancement was modifying the tracrRNA, the trans-activating crRNA component of the CRISPR Cas9 complex, to greatly improve the residency time for the Cas 9 complex, increasing the efficiency of the enzyme while lowering the loss of guide RNA during a screen^88,89^.

CRISPR has also been used to investigate non-coding regions of genomes and their impacts on gene regulation and drug resistance^90,91^. Pooled CRISPR Cas9 sgRNA libraries were designed to target non-coding regions surrounding three genes, *NF1*, *NF2*, and *CUL3*, which play a role in BRAF inhibitor (Vemurafenib) resistance in melanoma. A screen was performed to select for sgRNAs (targeting non-coding RNAs within their genomic region of interest) that would lead to resistance to BRAF inhibitor. The sgRNAs that conferred BRAF inhibitor resistance mapped to regions clustered around the *CUL3* gene. Analysis of the binding sites for sgRNAs led to the identification of putative transcription factor binding sites in the promoter of the *CUL3* gene. Transcription factor recruitment to the implicated *CUL3* regulatory sequences was analyzed by ChIP (chromatin immunoprecipitation analysis) in the presence and absence of sgRNAs. It was found that recruitment of transcription factors YY1, FOS and JUN required their cognate binding sites for *CUL3* promoter recruitment and gene regulation. *CUL3* gene expression was drastically decreased when the transcription factor binding sites were targeted by sgRNAs^90,91^.

Another CRISPR screen was performed in a non-small cell lung cancer model that harbored KRAS and homozygous mutant p53^10^. Cells were transduced with lentiviral sgRNAs and Cas9^10^. The transduced cells were propagated for one week and were then subcutaneously transplanted into the flanks of immunocompromised mice. At 6 weeks post-transplantation, the mice were sacrificed and the lungs were examined. None of the control mice had metastases whereas 8 out of the 9 injected mice had 80% of their lung lobes positive for metastasis^10^. The representation of guide RNAs in the tumors was examined by deep sequencing. Not surprisingly, one of the most commonly targeted genes in the lung metastases was the tumor suppressor Pten^10^. Additional genes were identified in the screen such as *NF1* and TRIM72^10^.

Yet another genome wide CRISPR screen was performed to identify cellular components that are required for ATR inhibitor sensitivity^92^. ATR inhibitors disable DNA repair mechanisms leading to replication stress and premature mitotic entry; eventually cells have mitotic catastrophe and undergo apoptosis^93,94^. Loss of *CDC25* by CRISPR Cas9 targeting led to resistance to ATR inhibitors^92^. CDC25 is a phosphatase that removes a key phosphate on mitotic cyclin dependent kinases to activate their function and promote cell cycle progression into mitosis^95^. Loss of CDC25 hindered early mitotic entry (normally observed with ATR inhibitor) and subsequent lesions to DNA. Interestingly inhibition of WEE1 led to sensitivity to ATR inhibitors^92^. WEE1 is the kinase that phosphorylates mitotic cyclin dependent kinases to prevent their activity and mitotic entry^96^. This high throughput CRISPR screen revealed constraints placed on mammalian systems that determine ATR inhibitor sensitivity^92^.

Some of the challenges involved in large-scale sensitivity screening using the CRISPR Cas9 system include dealing with polyploidy and aneuploidy in certain cancer cell lines, and the limits of screening for mutants of the cells and the detection of sgRNAs.

## 5. Ethical Issues of CRISPR Technology

The powerful utility of CRISPR Technology is not without ethical implications. Patent disputes have emerged in the United States, Europe and China^97-107^. Concerns about CRISPR use in agriculture and medical treatments have been raised^*^105,108-113^*^. Is CRISPR use safe in agricultural and medical settings? Scientists are only beginning to fully appreciate the capabilities as well as the ramifications of CRISPR technology.

Patent disputes between CRISPR application pioneers Dr. Zhang (Broad Institute) and Drs. Dounda and Charpentier (UC Berkeley and Max Planck Institute for Infection Biology) are ongoing^97,98^. Drs. Doudna and Charpentier published the design and use of hybrid sgRNAs with Cas9 to target genes in 2012^29^. Dr. Zhang published in 2013 the use of CRISPR in human and mammalian cells^11^. Of note, another scientist was also critical to the development of CRISPR technology applications. Dr. George Church (Harvard Medical School) published using CRISPR as a genome editing tool in human and mammalian cells in the same issue of Science as Dr. Zhang in 2013^28^. In addition to the initial implementation of CRISPR as a genome editing tool, numerous patents are pending in China for knocking out specific genes^105^. Chinese patents might also be applied to synthetic proteins as well as transgenic microorganisms^105^.

Chief among CRISPR associated ethical issues is human genetic engineering^113^. CRISPR has been employed in China to fight lung cancer and to mutate the β globin gene (*HBB*)^114,115^. The *PD1* gene was mutated using CRISPR in T cells isolated from a lung cancer patient^115^. PD1 is a death receptor found on T cells^116^. When bound by ligand, PD1 signal transduction negatively regulates T cell response. Loss of *PD1* by CRISPR mutagenesis led to increased T cell immunological responses such as cytotoxicity and INFγ production^115^. The genetically modified human T cells were cultured and re-introduced into the patient to enable the immune system to better mount a response to fight the cancer^115^. This scheme could be promising as antibodies that inhibit PD1 have had success in treating lung cancer in clinical trials^117,118^. Knowing that off-target effects are commonplace with CRISPR, genome editing strategies for immune cells is not without risk.

Another study employed CRISPR to mutate the *HBB* gene in tripronuclear human zygotes^114^. With *in vitro* fertilization technology, sometimes one egg will be fertilized with two sperm leading to tripronuclear zygotes. These zygotes will divide until they form a small clump and will then stop dividing. The tripronuclear cells were chosen as a model setting to study human genomic engineering with CRISPR. The human beta globin gene (*HBB*) is found mutated to a form that leads to sickle cell anemia, a homozygous recessive disease^119^. Millions of people world-wide harbor mutations in the *HBB* gene that changes the coding of amino acid 6 from a glutamic acid to a valine leading to protein aggregates and sickle shaped red blood cells^120^. Experiments performed by Liang *et al.* attempted to repair the mutated sickle alleles using CRISPR and HDR^114^. The repair of mutant *HBB* alleles in the human cells was relatively low^114^. Another observed pitfall was that injected zygotes displayed mosaicism for repair^114^. Potentially even more alarming was that off-target effects of CRISPR targeting were commonly observed in the zygotes^114^. This study highlighted the pitfalls of employing CRISPR as a genome edited tool in humans. More work must be done to ensure that genome edits are specific and efficient. It is important to point out that researchers have just recently reported that *HBB* mutations can be repaired in hematopoietic stem cells derived from human sickle cell anemia patients^121^.

While human cells and zygotes are being modified by CRISPR, scientists are still debating the ethical issues that are associated with using CRISPR in humans^105,113^. The use of CRISPR could improve outcomes for people who harbor mutations that lead to devastating diseases such as Alzheimer’s Disease and cancer^105,113^. However, the unintended consequence of using CRISPR must be considered. Off-target mutations could prove catastrophic leading to major birth defects, developmental deficiencies and cancer. The safety of employing this technology in modifying human genetics must first be rigorously explored before implementation can be considered.

Genetic engineering with CRISPR is taking agriculture by storm^122^. This technology is now being employed to make resistant crops, cattle without horns and pigs without disease^17,123,124^. How does one label genetically modified foods that were treated with CRISPR? Are these methods safe and ethical? Could these methodologies lead to needless suffering in animals that are experimental subjects? Can this technology become dangerous and propagated from animals to humans? Could sgRNAs for genetic modification in agriculture impact humans or the environment in an unforeseen way some day? These potential dangers stemming from CRISPR generated genetically modified foods should be rigorously explored before wide use.

## 6. Future Directions for CRISPR Technology

CRISPR Cas9 technology has massive potential to revolutionize biological research. Despite major breakthroughs since its discovery, CRISPR Cas9 is still a new frontier in genome engineering^59-61,63^. This technology dramatically expanded the ability to manipulate genes and many scientists recognize its potential to help understand and treat diseases. As the utilization of CRISPR Cas9 opens the door to build knowledge it has positive application in many fields of research. Its application in genome-wide studies will enable large-scale screening for drug treatments. It can be utilized in the agricultural research and pharmacological studies. Future research is directed to elucidate CRISPR Cas9 mode of action and improve the technology. A large focus on the improvement of CRISPR Cas9 will be on eliminating any off-target effects. This will include engineering or identifying smaller distinct Cas9 variants that may be more receptive to delivery in human cells. It is likely that it will be many years before CRISPR Cas9 is used to directly edit human genomes.

When contemplating the function of repetitive and viral sequences in prokaryotic defense from foreign DNA, one must wonder what the similar sequences in eukaryotes remain to reveal^125^. Are any of the viral sequences in humans hiding host defense mechanisms (similar to CRISPR) that will someday be discovered? Ironically, CRISPR technology is our best bet for uncovering the functionality of viral sequences found in the human genome (and many other genomes).

CRISPR technology will greatly progress knowledge about developmental processes and other basic biological mechanisms. As this technique becomes more commonplace and affordable, CRISPR use will be a staple in reverse genetic, forward genetic and basic cell biological investigations

## 7. Conclusions

In just three years since the first publications that utilized heterologous CRISPR Cas9 as a genome editing tool, it can already be seen that this technique has drastically increased research capabilities and molecular biological applications in many systems from deleting genes in cancer cell lines to genetically modifying plants^59-61,63^. Initially characterized as an adaptive immune response in Archaea and bacteria, this elegant molecular machinery was quickly adapted for use in many systems. CRISPR has ushered in an exciting time for science that is ripe for discovery.

## Bullet Points

**◊** **CRISPR endogenously provides adaptive immunity to many prokaryotes**

**◊ Heterologous CRISPR is a powerful genome editing tool that has revolutionized molecular biology**

**◊ Novel applications of CRISPR technology include lineage tracing, mRNA tracking and investigating epigenetics**

**◊** **Employing CRISPR to treat human disease is a major ethical issue**
